# Pooled Incidence of Heparin-Induced Thrombocytopenia and Anti-Platelet Factor 4 Antibody Formation Among Adults: A Systematic Review and Meta-Analysis

**DOI:** 10.7759/cureus.98710

**Published:** 2025-12-08

**Authors:** Anudeep Devarapalli, Ramya Rachamanti, Mounika Singampalli, Kiran Hullur

**Affiliations:** 1 Acute Medicine, Darlington Memorial Hospital, Darlington, GBR; 2 Pharmacology, Guntur Medical College, Guntur, IND; 3 General Medicine, Darlington Memorial Hospital, Darlington, GBR; 4 Respiratory Medicine, Darlington Memorial Hospital, Darlington, GBR

**Keywords:** heparin-induced thrombocytopenia, low-molecular-weight heparin, meta-analysis, platelet factor 4 antibodies, seroconversion, unfractionated heparin

## Abstract

Heparin-induced thrombocytopenia (HIT) is an immune-mediated drug reaction. Patients with HIT show thrombocytopenia and increased thrombotic risk. Unfractionated heparin (UFH) is more immunogenic compared to low-molecular-weight heparin (LMWH), but estimates vary across clinical settings. The study aimed to determine the pooled incidence of thrombocytopenia and anti-platelet factor (PF)-4 antibody formation among adults exposed to UFH and LMWH. We systematically searched electronic databases, including PubMed, Cochrane CENTRAL, Embase (via Cochrane), the International Clinical Trials Registry Platform (ICTRP), and ClinicalTrials.gov, for studies published from 2010 to 2025. Eligible studies reported clinical HIT incidence or anti-PF4/heparin antibody formation in adults receiving UFH or LMWH. Two reviewers independently performed screening and data extraction. Bias risk was assessed using the Risk Of Bias In Non-randomised Studies - of Interventions (ROBINS-I). Random-effects models pooled risk ratios (RRs) comparing UFH with LMWH. Twelve studies comprising 139,744 adults were included. Six studies reporting clinical HIT showed more risk with UFH (RR 3.4; I^2^ = 55%). Four studies done on antibody formation showed higher seroconversion with UFH (RR 2.2; I^2^ = 45%). The certainty of evidence (assessed using the Grading of Recommendations Assessment, Development and Evaluation (GRADE) framework) was low due to observational designs, diagnostic heterogeneity, and imprecision. UFH is associated with a higher incidence of clinical HIT and anti-PF4/heparin antibody formation compared with LMWH. Though certainty is low, the findings support using LMWH when clinically appropriate.

## Introduction and background

Heparin-induced thrombocytopenia (HIT) is an immune-mediated adverse reaction characterized by thrombocytopenia and a paradoxical increase in thrombotic risk. It develops when IgG antibodies form against platelet factor 4 (PF4) bound to heparin, leading to platelet activation through FcγRIIa receptors and resulting in accelerated platelet consumption and thrombin generation [1-3]. Because heparin is routinely used in postoperative care in India, understanding the true burden of anti-PF4 antibody formation (immunogenicity) and clinically confirmed HIT is essential for improving risk stratification and guiding treatment decisions.

HIT is clinically significant due to its consequences, such as deep-vein thrombosis (DVT), pulmonary embolism, limb ischemia, and myocardial infarction. Patients previously exposed to heparin may develop symptoms rapidly, making early recognition critical for preventing thrombotic complications. Despite improvements in diagnostic pathways, variations in assay availability, clinician familiarity with HIT scoring systems, and differences in pre-test probability assessment can contribute to under- and over-diagnosis. Reliable pooled evidence on immunogenicity and clinical HIT incidence may help strengthen diagnostic stewardship and optimize anticoagulant selection in high-risk settings [1-3].

Although many studies have compared rates of anti-PF4 antibodies and HIT between unfractionated heparin (UFH) and low-molecular-weight heparin (LMWH), their findings are inconsistent. Biological factors likely contribute to this variability, as UFH has a greater molecular size and stronger PF4 binding affinity, leading to the formation of larger, more immunogenic complexes [4,5]. Clinical diagnosis is further complicated by the fact that thrombocytopenia in hospitalized patients often has multiple potential causes and may be affected by differences in laboratory assays [6]. Studies in cardiac surgery populations have shown high rates of postoperative anti-PF4/heparin antibody seroconversion, reflecting the large heparin exposures typically used in this setting [7]. An earlier pooled analysis published in 2005 has demonstrated a higher incidence of HIT with UFH compared with LMWH across surgical and medical populations [8].

Evidence suggests that trauma severity affects PF4-heparin immune responses, with more extensive tissue injury associated with increased antibody formation and a higher risk of HIT [9]. Clinical guidance highlights HIT as a serious and potentially fatal complication of heparin therapy, underscoring the importance of rapid recognition and the use of standardized diagnostic tools such as the Thrombocytopenia, Timing, Thrombosis, and Other causes (4Ts) score, followed by confirmatory functional assays when available [10]. The last pooled analysis on this topic was from 2005. Other analyses differed from our objective. So, the current meta-analysis was done.

Objective

The objective of this study is to determine the pooled incidence of thrombocytopenia and anti‑PF4 antibody formation among adults exposed to UFH and LMWH.

## Review

Methodology

This systematic review and meta-analysis were done as per the Preferred Reporting Items for Systematic Reviews and Meta-Analyses (PRISMA) guidelines. The PRISMA checklist was attached in Appendix A. The review aimed to synthesize contemporary evidence on the incidence of HIT and anti-PF4 antibody formation among adults receiving UFH or LMWH.

Eligibility Criteria

Inclusion criteria: Studies were eligible if they included adult participants aged 18 years or older and evaluated exposure to UFH or LMWH. Eligible studies were required to report clinically adjudicated HIT, or anti-PF4/heparin antibody positivity determined by enzyme-linked immunosorbent assay (ELISA), serotonin release assay (SRA), or other validated functional assays. Only studies that provided extractable data on the number of events and total participants in each heparin exposure group were included. Publications had to be available in English and published between 2010 and 2025.

Exclusion criteria: Case reports, case series, reviews, editorials, and conference summaries, studies involving children or pregnant populations were excluded. Studies that did not provide clearly separable UFH and LMWH data, non-human studies, in-vitro experiments, and those lacking extractable outcome measures were also excluded.

Search Strategy

A thorough literature search was done among PubMed, Cochrane CENTRAL, Embase (through Cochrane interface), the International Clinical Trials Registry Platform (ICTRP), and ClinicalTrials.gov from January 1, 2010, to November 1, 2025. The PubMed search used the following terms: “heparin-induced thrombocytopenia” OR “HIT” OR “anti-PF4 antibodies” AND “heparin.” Filters were applied for human subjects, adults, and the English language. Equivalent syntax was adapted for other databases to ensure consistency.

Selection of Studies

**Figure 1:**
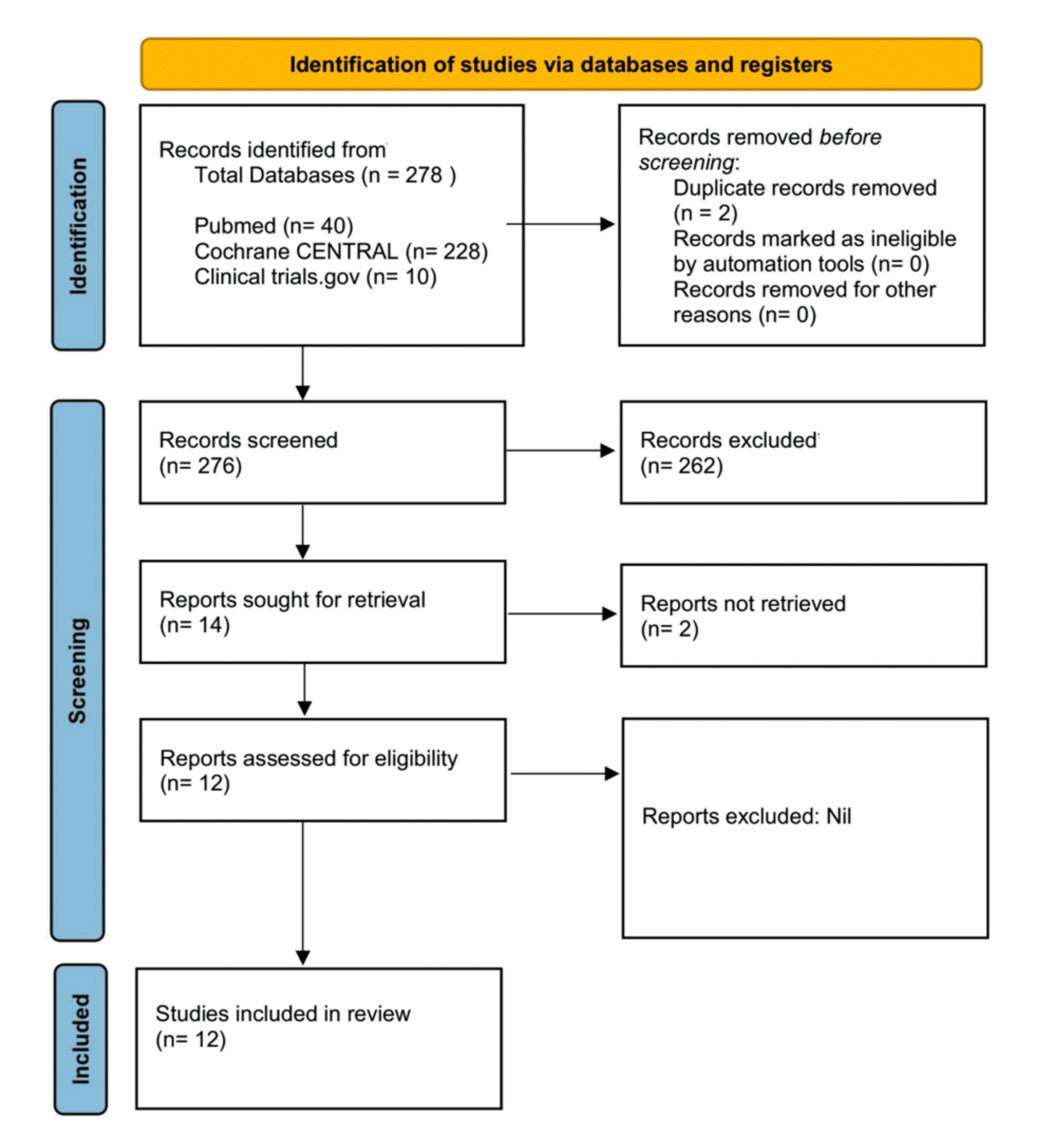
PRISMA flow diagram illustrating the identification, screening, and inclusion of 12 studies. PRISMA: Preferred Reporting Items for Systematic Reviews and Meta-Analyses; CENTRAL: Cochrane Controlled Register of Trials

Two reviewers screened titles and abstracts, and then, the full-texts of relevant articles. Discrepancies were solved through discussion, and if there is still disagreement, the third reviewer's opinion is taken. Forty PubMed records and additional records from Embase, Cochrane CENTRAL, and clinicaltrials.gov underwent screening, after which 12 studies met all eligibility criteria and were included in the quantitative synthesis, as shown in Figure 1. Full search strategies are provided in Appendix B.

Data Extraction

Data extraction was done using a standardized form. Parameters included were study author, year, design, setting, sample size, age, sex distribution, UFH or LMWH exposure, the number of anti-PF4 antibody-positive participants, and the number of clinically verified HIT events.

Measured Outcomes

Primary outcome: The incidence of clinically adjudicated HIT among patients exposed to UFH versus LMWH is the main outcome. 

Secondary outcome: The secondary outcome was the incidence of anti-PF4 antibody seroconversion, defined by assay-specific thresholds (most commonly ELISA optical density cutoff values) or functional platelet-activation tests.

HIT

HIT was defined as a decrease in platelet count associated with heparin exposure, supported by confirmatory clinical or laboratory criteria as reported in each included study.

Assessment of Methodological Quality

Risk of bias was evaluated using the Risk Of Bias In Non-randomised Studies - of Interventions (ROBINS-I) tool, which assessed seven domains. They include confounding, participant selection, exposure classification, deviations from intended exposure, missing data, measurement of outcomes, and reporting bias.

Data Synthesis

Risk ratios (RRs) were calculated for each study by comparing UFH and LMWH groups on the log scale and back-transformed. A DerSimonian-Laird random-effects model was used to pool study-level RRs, due to expected heterogeneity in study settings and populations. Statistical heterogeneity was assessed using Cochran’s Q, τ^2^, and Higgins’ I^2^ statistics. Sensitivity analyses were performed by excluding individual studies (leave-one-out analysis). Analyses were done using R version 4.5.2 (R Foundation for Statistical Computing, Vienna, Austria) [11], employing the metafor, meta, dmetar, and robvis packages for meta-analysis and risk-of-bias visualization.

Results

Literature Search and Study Selection

The search strategy found 40 records from PubMed and studies through CENTRAL and Embase. After the eligibility assessment, 12 studies met all inclusion criteria and were incorporated into the final quantitative synthesis.

Characteristics of Included Studies

Across the 12 studies, 139,744 adult patients were evaluated. The study settings included orthopedic surgery cohorts, cardiac surgery patients, hemodialysis populations, critically ill medical patients, and large national registry datasets. Table 1 outlines the characteristics of the included studies. Twelve studies were included. They represent diverse clinical populations across cardiac surgery, orthopedic surgery, venous thromboembolism registries, hospitalized medical cohorts, and laboratory-based evaluation. Study designs ranged from prospective and retrospective cohorts to multicenter and registry-based datasets. Sample sizes vary widely from 38 to 63,561 patients. Most studies involved adult or elderly populations, with mean or median ages between 49 and 76 years. The proportion of female participants ranged from 25.6% to 84%. Overall, 47.7% of participants were female (Table 1). Other details of study characteristics, such as study location, study design, etc., were mentioned in Table 2.

**Table 1:** Characteristics of the 12 included studies, including design, sample size, population type, age distribution. NR: not reported

Study	Design	Sample Size	Age (Years)	Female (%)
Welsby et al., 2017 [7]	Cardiac surgery cohort	946	62	25.6
Lubenow et al., 2010 [9]	Prospective multicenter	614	49-50	43
Griffin et al., 2012 [12]	Prospective observational	100	76.3	55
Falvo et al., 2011 (RIETE) [13]	Registry study	24,401	NR	48.7
Motokawa et al., 2011 [14]	Prospective orthopedic cohort	374	66-74	80-84
Bito et al., 2016 [15]	Multicenter cohort	462	NR	83.7
Selleng et al., 2015 [16]	Prospective observational	320	NR	NR
Van Matre et al., 2018 [17]	Retrospective cohort	63,561	NR	NR
Al-Eidan, 2015 [18]	Retrospective cohort	46,638	64	42
Linkins et al., 2015 [19]	Prospective cohort	526	66.5	48.6
Nilius et al., 2024 [20]	Observational	147	68.5	35.7
Železnik et al., 2021 [21]	Laboratory-based cohort	38	74.8	55

**Table 2:** Key methodological characteristics of the included studies. HIT: heparin-induced thrombocytopenia; PF4: platelet factor 4; CPB: cardiopulmonary bypass; RCT: randomized controlled trial; VTE: venous thromboembolism; THA/TKA: total hip arthroplasty/total knee arthroplasty; UFH: unfractionated heparin; LMWH: low-molecular-weight heparin; OD: optical density threshold for ELISA positivity; ELISA: enzyme-linked immunosorbent assay; SRA: serotonin release assay; HIPA: heparin-induced platelet activation test; HNA: heparin neutralization assay; PIFT: platelet immunofluorescence test; NR: not reported

Study	Country	Population	Study Design	Study Period	Heparin Type	HIT/Anti-PF4 Definition	Diagnostic Assay	Outcomes Measured
Welsby et al., 2017 [7]	USA	Adults undergoing cardiac surgery with CPB	Multicenter prospective cohort	2006-2014	UFH (intraop), some postop heparin	OD>0.4/1.0 + confirmatory inhibition	GTI ELISA; SRA subset	Seroconversion; 90-day outcomes
Lubenow et al., 2010 [9]	Germany	Trauma ≥5 days prophylaxis	Double-blind RCT	2003-2005	UFH vs. LMWH certoparin	Seroconversion + HIPA-positive HIT	In-house EIA; HIPA	Seroconversion; HIT
Griffin et al., 2012 [12]	Denmark	Hip fracture	Lab analysis of RCT samples	PK-532	UFH vs. enoxaparin	OD>0.40	GTI ELISA; isotypes	Anti-PF4 prevalence
Falvo et al., 2011 (RIETE) [13]	Multinational	VTE registry	Prospective cohort	2001-2009	UFH/LMWH	Platelets ≤150K after ≥1 day	Platelet count	HAT 6-month incidence
Motokawa et al., 2011 [14]	Japan	THA/TKA	Prospective cohort	2006-2010	UFH/LMWH/fondaparinux	IgG OD≥0.40 + platelet fall	GTI ELISA; platelet count; imaging	Seroconversion; thrombosis
Bito et al., 2016 [15]	Japan	Elective TKA/THA	Multicenter cohort	NR	UFH/LMWH/fondaparinux	POD10 seroconversion OD≥1.4	Asserachrom HPIA ELISA	Seroconversion by regimen
Selleng et al., 2015 [16]	NR	NR	Prospective observational	NR	NR	NR	NR	NR
Van Matre et al., 2018 [17]	USA	ICU adults with cancer	Retrospective administrative cohort	2010-2014	Enoxaparin vs. UFH	ICD-9 HIT	Administrative	New DVT; HIT incidence
Al-Eidan, 2015 [18]	Saudi Arabia (KAMC Riyadh)	Adults ≥18 yrs receiving UFH/LMWH	Retrospective cohort	2011-2013	UFH; enoxaparin	≥50% fall + 4Ts + ELISA OD>0.4	Asserachrom HPIA ELISA	Annual HIT incidence; UFH vs. LMWH RR
Linkins et al., 2015 [19]	Canada (four hospitals)	Suspected HIT patients	Prospective cohort	2008-2013	UFH, LMWH, both	SRA>50% + inhibition + IgG EIA>0.70	PF4/H-PaGIA; SRA; IgG EIA	Mgmt failures; diagnostic performance; outcomes
Nilius et al., 2024 [20]	Switzerland, Germany, USA	Suspected HIT; 3 groups: HIPA+ HIT; ELISA+ HIPA-; ELISA- HIPA-	Prospective cohort (TORADI-HIT proteomic study)	TORADI-HIT recruitment period	Heparin exposure implied in suspected HIT	HIT = positive HIPA; antibody+ = ELISA>0.5 with negative HIPA; HIT- = ELISA- + HIPA-	HIPA; lifecodes PF4 enhanced ELISA; Olink PEA proteomics; P-selectin ELISA	Proteomic biomarker discovery; ROC AUC; P-selectin levels; thrombosis associations
Železnik et al., 2021 [21]	Slovenia	38 suspected HIT patients, ELISA-positive, heparin-treated; medical and surgical	Retrospective laboratory cohort	NR	Heparin-treated patients (UFH/LMWH not individually specified)	ELISA IgG OD>0.4 (Immucor GTI, PF4-PVS); HNA ≥50% inhibition; platelet-activating antibodies = >10% CD62P+ at low heparin + ≥50% inhibition at high heparin	IgG ELISA; heparin neutralization assay; in-house PIFT; in-house flow cytometric activation assay (CD61/CD62P)	Platelet activation across three OD groups; correlation of OD with % activated donors; presence of other platelet antibodies; characterization of ELISA/PIFT/flow results

Clinical HIT Incidence (UFH vs. LMWH)

**Figure 2:**
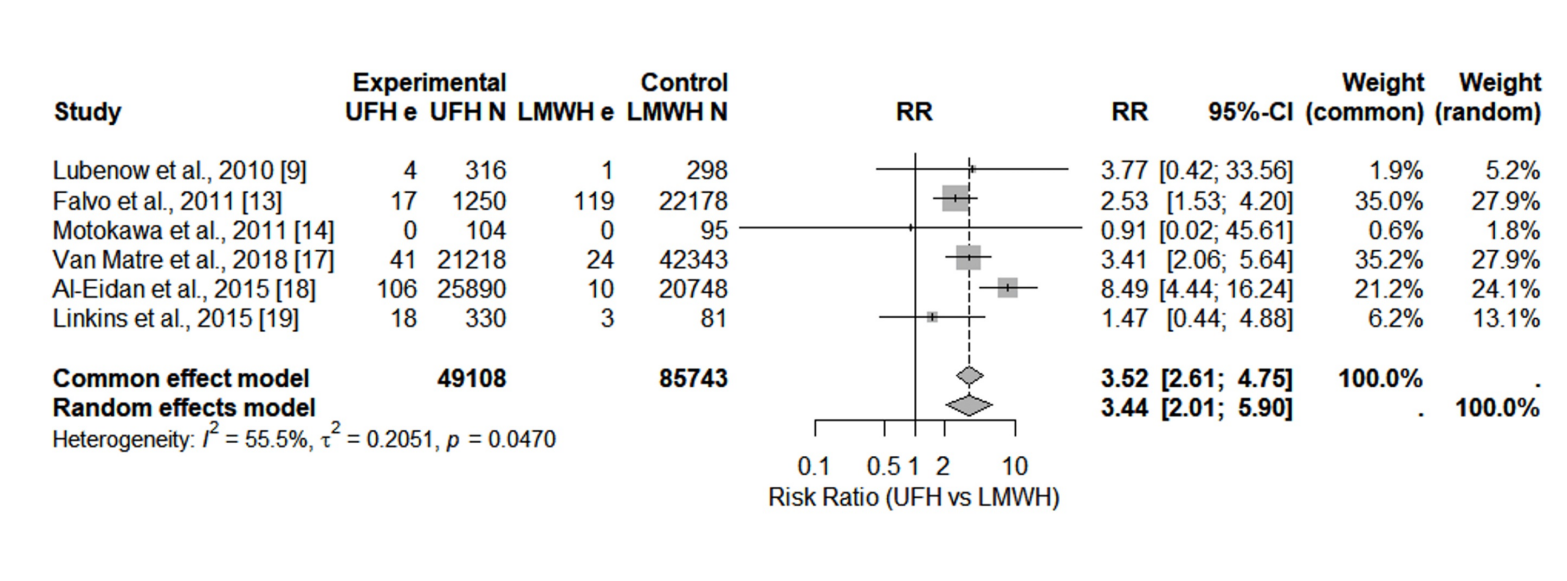
Forest plot showing the pooled risk ratio of HIT (UFH vs. LMWH). HIT: heparin-induced thrombocytopenia; UFH: unfractionated heparin; LMWH: low-molecular-weight heparin; RR: risk ratio; CI: confidence interval References [9,13,14,17-19]

Six studies reported clinically adjudicated HIT or registry-recorded heparin-associated thrombocytopenia (HAT) events for UFH and LMWH. All individual effect estimates favored a higher HIT risk associated with UFH. The pooled random-effects RR was 3.4, showing a 3.4-fold increased risk of HIT with UFH. Moderate heterogeneity was observed (I^2^ = 55.5%). Figure 2 presents the forest plot.

**Figure 3:**
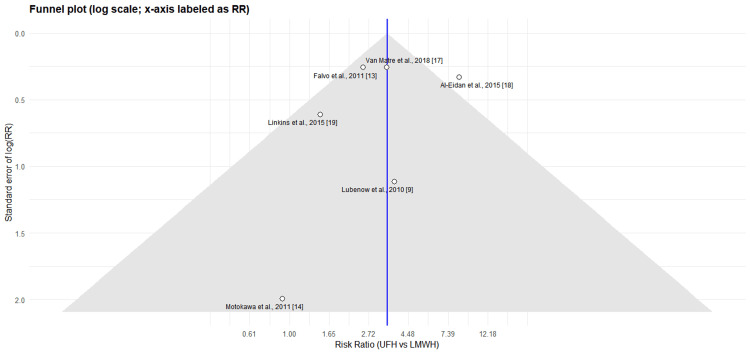
Funnel plot showing the publication bias in studies of HIT incidence. HIT incidence studies: Falvo et al., 2011 [13]; Van Matre et al., 2018 [17]; Lubenow et al., 2010 [9]; Al-Eidan, 2015 [18]; Motokawa et al., 2011 [14]; Linkins et al., 2015 [19] HIT: heparin-induced thrombocytopenia; UFH: unfractionated heparin; LMWH: low-molecular-weight heparin; RR: risk ratio

Sensitivity analyses, performed by removing individual studies sequentially, demonstrated consistent pooled estimates with no influential outliers (Table 3). Publication bias was assessed using funnel plots (Figure 3).

**Table 3:** Leave-one-out sensitivity analysis evaluating the influence of each individual study on the pooled RR for clinical HIT incidence. RR: risk ratio; CI: confidence interval; HIT: heparin-induced thrombocytopenia

Study Removed	Pooled RR	95% CI
Falvo et al., 2011 [13]	3.72	2.08-6.66
Van Matre et al., 2018 [17]	3.19	1.82-5.60
Lubenow et al., 2010 [9]	3.48	2.01-6.01
Al-Eidan, 2015 [18]	2.62	1.66-4.14
Motokawa et al., 2011 [14]	3.53	2.06-6.04
Linkins et al., 2015 [19]	3.64	2.09-6.32

Anti-PF4 Antibody Seroconversion

**Figure 4:**
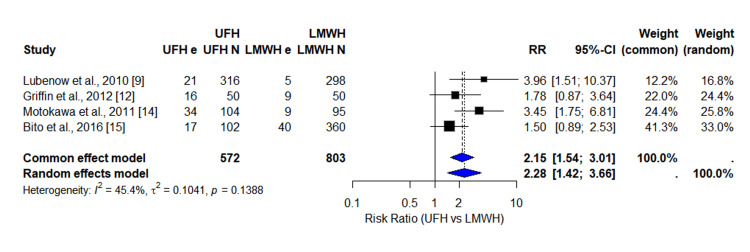
Forest plot showing the pooled risk ratio of PF4 antibody positivity status (UFH vs. LMWH). UFH: unfractionated heparin; LMWH: low-molecular-weight heparin; RR: risk ratio; CI: confidence interval References [9,12,14,15]

Four studies reported comparable seroconversion data for UFH and LMWH. The pooled RR for anti-PF4 antibody formation was 2.28 (95% confidence interval (CI): 1.42-3.66), indicating more than double the risk of seroconversion among patients receiving UFH. Heterogeneity was moderate (I^2^ = 45.4%). Figure 4 shows a Forest plot for the pooled relative risk.

**Figure 5:**
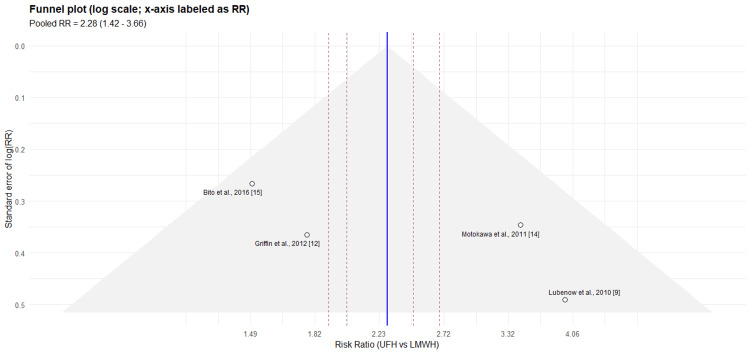
Funnel plot showing the publication bias (antibody positivity studies). Antibody status reported studies: Bito et al., 2016 [15]; Griffin et al., 2012 [12]; Motokawa et al., 2011 [14]; Lubenow et al., 2010 [9] UFH: unfractionated heparin; LMWH: low-molecular-weight heparin; RR: risk ratio

Sensitivity analysis for antibody positivity (Table 4) confirmed stable pooled estimates. Funnel plot inspection showed possible small-study effects (Figure 5).

**Table 4:** Leave-one-out sensitivity analysis assessing the impact of individual study exclusion on the pooled RR for PF4 antibody seroconversion. PF4: platelet factor 4; RR: risk ratio; CI: confidence interval

Study Removed	Pooled RR	95% CI
Bito et al., 2016 [15]	2.68	1.68-4.26
Griffin et al., 2012 [12]	2.47	1.35-4.51
Motokawa et al., 2011 [14]	1.90	1.20-3.01
Lubenow et al., 2010 [9]	2.01	1.25-3.22

Risk of Bias Assessment

**Figure 6:**
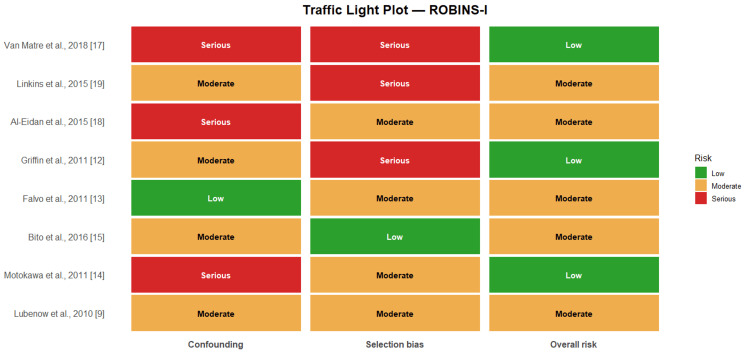
Traffic light plot using the ROBINS-1. Green: Low risk; Orange: Moderate risk; Red: Critical risk; ROBINS-I: Risk Of Bias In Non-randomised Studies - of Interventions References [9,12-15,17-19]

ROBINS-I evaluation showed moderate risk of bias in most observational studies and serious risk in a minority, largely due to confounding, measurement variability, and exposure misclassification (Table 5). Overall certainty using the Grading of Recommendations Assessment, Development and Evaluation (GRADE) was rated low for both primary outcomes due to inconsistency and study design limitations. The GRADE summary and Robin's traffic light plot are provided in Table 6 and Figure 6, respectively.

**Table 5:** Risk of bias assessment using the ROBINS-I. Low: Comparable to a well-conducted randomized trial; Moderate: Sound for a non-randomized study but with some limitations; Serious: Important problems that may substantially lower confidence in the results; Critical: Too problematic to provide reliable evidence (none in this dataset). ROBINS-I: Risk Of Bias In Non-randomised Studies - of Interventions

Study	Confounding	Selection Bias	Overall Risk
Lubenow et al., 2010 [9]	Moderate	Low	Moderate
Griffin et al., 2011 [12]	Serious	Moderate	Serious
Falvo et al., 2011 [13]	Moderate	Low	Moderate
Motokawa et al., 2011 [14]	Moderate	Low	Moderate
Bito et al., 2016 [15]	Moderate	Low	Moderate
Van Matre et al., 2018 [17]	Serious	Moderate	Serious
Al-Eidan, 2015 [18]	Serious	Moderate	Serious
Linkins et al., 2015 [19]	Moderate	Low Moderate	Moderate

**Table 6:** GRADE evidence profile. High: Very confident in the effect estimate; Moderate: Moderately confident (true effect likely close); Low: Limited confidence (true effect may differ more); Very Low: Very little confidence (true effect is uncertain) RR: risk ratio; CI: confidence interval; HIT: heparin-induced thrombocytopenia; UFH: unfractionated heparin; LMWH: low-molecular-weight heparin; GRADE: Grading of Recommendations Assessment, Development and Evaluation

Outcome	Effect, RR (CI)	No. of Participants	Certainty	Comments
Clinical HIT (UFH vs. LMWH)	RR 3.44 (2.01-5.90)	≈139,744	Low	Observational designs + heterogeneity
Anti-PF4 Antibody Seroconversion	RR 2.29 (1.42-3.69)	1,375	Low	Assay heterogeneity, small sample size
Major Bleeding	Inconsistent	Few studies	Very Low	Sparse data
HIT-Related Mortality	Rare	Very limited	Very Low	Insufficient events

Discussion

This meta-analysis, which included 12 studies from a variety of clinical settings, found that UFH carried a higher risk of anti-PF4 antibody formation and clinically confirmed HIT when compared with LMWH. The difference between these agents aligns with the established immunologic behavior of UFH, whose longer polysaccharide chains and stronger binding affinity for PF4 promote the formation of larger antigenic complexes capable of triggering IgG-mediated platelet activation [4,5]. These mechanisms have been well described in earlier literature and remain supported by more recent clinical investigations [1-3].

The included studies represented diverse patient populations - orthopedic surgery, cardiac surgery, dialysis, cancer cohorts, and critically ill medical patients [12,14,17]. This heterogeneity provides context for the moderate variability seen in the pooled estimates (I^2^ ≈ 56%). Clinical circumstances affect the chance of seroconversion; for example, cardiac surgery and hemodialysis expose patients to large or repeated doses of UFH, which has long been associated with higher anti-PF4 antibody production [7,12]. LMWH-based prophylaxis used in orthopedic and general medical settings consistently showed lower rates of seroconversion and HIT, in line with earlier epidemiological evidence [9,10]. As part of the methodological appraisal framework for included studies, eligibility criteria were aligned with the domains recommended by the ROBINS-I tool [22].

Our pooled antibody seroconversion analysis showed that UFH was associated with more than double the risk of developing anti-PF4 antibodies. However, antibody positivity alone does not always translate into clinical HIT, as many patients may have non-pathogenic antibodies that do not activate platelets or cause thrombosis. This difference was proven in laboratory studies and clinical cohorts where ELISA positivity exceeded functional platelet activation [6,23]. For this reason, confirmatory testing, particularly the SRA, is essential for accurate diagnosis.

The underlying biology of HIT extends beyond platelet activation. PF4/heparin complexes can interact with endothelial cells and monocytes, leading to tissue factor expression and enhanced thrombin generation, thereby driving thrombosis rather than isolated thrombocytopenia. These processes help explain the significant thrombotic burden associated with HIT [23-25]. Individual inflammatory states, such as those seen in trauma, major surgery, and critical illness, may heighten PF4 release or alter immune responsiveness, contributing to the variability observed in seroconversion across different populations [26]. HIT risk is shaped not only by the type of heparin used but also by the patient’s immunologic and inflammatory environment at the time of exposure.

These mechanistic insights also reinforce the importance of careful diagnostic assessment. The 4Ts score remains the standard first step; combining the score with quantitative ELISA thresholds and functional assays can refine diagnostic certainty, especially in borderline cases [27]. In groups known to be at higher risk, such as cardiac surgery patients or those receiving repeated UFH exposures, early transition to LMWH when feasible, along with structured platelet monitoring, may help reduce adverse outcomes. Recent therapeutic approaches, including the use of intravenous immunoglobulin (IVIG) or plasma exchange in complex cases with persistent platelet-activating antibodies, represent evolving strategies that may benefit select patients, although more evidence is needed before routine adoption.

The findings of this review strengthen the growing evidence that the risk of HIT varies significantly between UFH and LMWH across diverse patient populations. Importantly, the variation seen across studies proves the impact of heterogeneity in diagnostic approaches. Some cohorts depended on ELISA testing alone [13,17,18], and others used functional assays and SRA apart from ELISA, which accurately identify clinically significant HIT [9,14,19]. This methodological inconsistency proves the need for standardized testing algorithms and uniform reporting of both immunologic and functional assay results. Another important observation is the clinically relevant difference between antibody positivity and clinically adjudicated HIT. Several studies reported either seroprevalence or confirmed HIT rates [9,13-15,17-19]. Only Motokawa et al.'s study [14] reported both, but found a seroconversion rate as high as compared to HIT incidence, proving that laboratory antibody detection alone may not serve as a surrogate for clinical diagnosis. So, clinicians must combine laboratory findings with validated scoring systems such as the 4Ts score, especially in populations with a high baseline risk of thrombocytopenia.

Though our findings favor LMWH as the safer option, especially in medical and postoperative patients, patients requiring cardiopulmonary bypass continue to depend on UFH. So, tailored prophylactic and surveillance strategies are needed rather than a universal shift away from UFH. Future studies should focus on prospective multicenter cohorts with harmonized diagnostic definitions, clearer separation of UFH and LMWH exposure groups, and standardized reporting of both antibody and clinical endpoints. These efforts would facilitate more accurate pooled estimates and strengthen evidence-based recommendations. As newer anticoagulants and heparin alternatives gain prominence, comparative studies assessing their HIT risk profile are also needed.

The primary limitation of this review stems from the predominance of observational studies, which inherently carry risks of confounding. UFH is frequently used in patients with renal impairment or severe conditions that can independently influence platelet counts. Additionally, thrombocytopenia is common in hospitalized patients for many unrelated reasons, making diagnostic consistency challenging when laboratory assays or institutional criteria vary. But the direction of effect is uniform across sensitivity analyses, indicating that the higher HIT risk associated with UFH is a robust finding. These results align with earlier pooled analyses that demonstrated substantially lower HIT risk with LMWH compared with UFH. The consistency between older and newer literature suggests that, in spite of improvements in diagnostic assays and clinical awareness, the inherent immunogenic difference between UFH and LMWH persists. Overall, the findings from this updated synthesis reinforce the preference for LMWH in settings where both agents are clinically acceptable, particularly when reducing HIT risk is a priority.

## Conclusions

This meta-analysis showed that UFH is associated with higher rates of HIT and anti-PF4 antibody seroconversion compared with LMWH. Across six studies reporting clinical HIT, the pooled RR showed that UFH carried a 3.41-fold higher risk with moderate heterogeneity. Four studies evaluating seroconversion showed a 2.2-fold likelihood of developing anti-PF4 antibodies among patients receiving UFH. Robustness of results across leave-one-study-out analyses and across varied clinical settings reinforces the reliability of these findings. Overall, the evidence indicates that UFH poses significantly greater immunogenic and clinical HIT risk. These results support prioritizing LMWH when clinically appropriate to reduce the likelihood of HIT-related complications.

## Appendices

Appendix A

**Table 7:** Overall PRISMA compliance (96%). PRISMA: Preferred Reporting Items for Systematic Reviews and Meta-Analyses

Section	PRISMA Item	Requirement	Status
TITLE	1	Identify as systematic review/meta-analysis	Available
ABSTRACT	2	Structured abstract following PRISMA-A	Available
INTRODUCTION	3	Rationale	Available
	4	Objectives	Available
METHODS	5	Protocol registration	Stated not registered
	6	Eligibility criteria	Available
	7	Information sources	Available
	8	Search strategy	Available in supplementary material
	9	Study selection process	Available
	10	Data extraction process	Available
	11	Data items	Available
	12	Risk of bias assessment	Available
	13	Effect measures	Available
	14	Synthesis methods	Available
	15	Heterogeneity reporting	Available
	16	Sensitivity analyses	Available
RESULTS	17	Study selection with PRISMA flowchart	Available (Figure 1)
	18	Study characteristics	Available (Table 1)
	19	Risk of bias	Available (Supplementary Table S1)
	20	Results of individual studies	Available (Tables 2–4)
	21	Meta-analysis results	Available
	22	Certainty of evidence	Available (Supplementary Table S2)
DISCUSSION	23	Interpretation	Available
	24	Limitations	Available
	25	Conclusions	Available
OTHER	26	Funding	Available
	27	Competing interests	Available
	28	Availability of data	All data extracted from published studies.

Appendix B

Full Search Strategies

PubMed Search Strategy (2010-2025)

("Heparin-Induced Thrombocytopenia"[Mesh]

OR "heparin-induced thrombocytopenia"[Title/Abstract]

OR "HIT"[Title/Abstract]

OR "heparin associated thrombocytopenia"[Title/Abstract]

OR "anti-PF4"[Title/Abstract]

OR "platelet factor 4"[Title/Abstract])

AND

(heparin[Title/Abstract] OR "unfractionated heparin"[Title/Abstract]

OR UFH[Title/Abstract]

OR "low molecular weight heparin"[Title/Abstract]

OR LMWH[Title/Abstract]

Filters applied: Humans; English; 2010-2024. Records retrieved: 40.

Finally selected from this: 12

Cochrane CENTRAL Search Strategy (2010-2025)

("heparin induced thrombocytopenia"

OR "heparin-induced thrombocytopenia"

OR HIT

OR "anti-PF4"

OR "platelet factor 4")

Search fields: Title, Abstract, Keywords. Years: 2010-2025.

Records retrieved: 228.

Finally selected: 2

Embase (via Cochrane) Search Strategy

('heparin induced thrombocytopenia'/exp

OR 'heparin induced thrombocytopenia'

OR HIT

OR 'heparin associated thrombocytopenia'

OR 'platelet factor 4'

OR 'platelet factor 4'

OR 'anti PF4'

OR 'anti-PF4')

AND

(heparin OR 'unfractionated heparin' OR UFH

OR 'low molecular weight heparin' OR LMWH

Limits applied: Humans; English; Adults; 2010-2024. Records retrieved: 77.

All these 77 are duplicates of above 268 studies

Finally selected from this: 0

ICTRP Search

Search term: "heparin-induced thrombocytopenia"

Filters: All countries; all recruitment statuses.

No eligible studies with extractable HIT incidence.

Finally selected from this: 0

Clinicaltrials.gov

Condition or disease: "heparin-induced thrombocytopenia"

Other terms: "HIT", "anti-PF4", "platelet factor 4"

Study type: All

Records retrieved: 10.

Finally selected from this: 0

Finally, all 278 studies were opened and assessed for eligibility.
